# Potential Use and Challenges of Functional Connectivity Mapping in Intractable Epilepsy

**DOI:** 10.3389/fneur.2013.00039

**Published:** 2013-05-22

**Authors:** Robert Todd Constable, Dustin Scheinost, Emily S. Finn, Xilin Shen, Michelle Hampson, F. Scott Winstanley, Dennis D. Spencer, Xenophon Papademetris

**Affiliations:** ^1^Department of Diagnostic Radiology, Yale School of MedicineNew Haven, CT, USA; ^2^Department of Neurosurgery, Yale School of MedicineNew Haven, CT, USA; ^3^Department of Biomedical Engineering, Yale UniversityNew Haven, CT, USA; ^4^Interdepartmental Neuroscience Program, Yale UniversityNew Haven, CT, USA

**Keywords:** epilepsy, functional connectivity, connectome, fMRI, network theory, graph theory, surgical planning

## Abstract

This review focuses on the use of resting-state functional magnetic resonance imaging data to assess functional connectivity in the human brain and its application in intractable epilepsy. This approach has the potential to predict outcomes for a given surgical procedure based on the pre-surgical functional organization of the brain. Functional connectivity can also identify cortical regions that are organized differently in epilepsy patients either as a direct function of the disease or through indirect compensatory responses. Functional connectivity mapping may help identify epileptogenic tissue, whether this is a single focal location or a network of seizure-generating tissues. This review covers the basics of connectivity analysis and discusses particular issues associated with analyzing such data. These issues include how to define nodes, as well as differences between connectivity analyses of individual nodes, groups of nodes, and whole-brain assessment at the voxel level. The need for arbitrary thresholds in some connectivity analyses is discussed and a solution to this problem is reviewed. Overall, functional connectivity analysis is becoming an important tool for assessing functional brain organization in epilepsy.

## Introduction

Functional connectivity in human neuroscience refers to the synchrony of activity in anatomically distinct regions of the brain: if two areas are highly correlated in their activity over time, they are considered functionally connected. As measured by fMRI, functional connectivity relies on the blood oxygenation level dependent (BOLD) contrast mechanism (Ogawa et al., 1990, 1992), the same as that used in traditional task-based functional MRI studies. But, rather than examining changes in response to specific input stimuli in a block design or event-related paradigm, connectivity mapping can extract information from correlations in the fMRI time-course data while the subject is at rest, in the absence of any externally imposed task. Acquiring connectivity data in the resting state makes connectivity analysis easily adaptable to clinical scanning as it requires no subject participation other than to remain still in the magnet and is thus just like any clinical imaging study. It can also be used intraoperatively in anesthetized patients.

Task-based fMRI provides exquisite maps of functional regions differentially involved in the execution of a specific task and is the foundation of most functional brain imaging research. Task-based fMRI as it is used in surgical planning in epilepsy is chiefly focused on identifying eloquent cortex that must be spared in a surgical intervention, while other techniques are used to identify the epileptogenic tissue to be resected. Task-based fMRI is not well suited for identifying abnormal cortical or subcortical tissue throughout the brain, because only a very small number of regions typically show differential activation to any given task and the number of tasks that can be run in a reasonable time with sufficient statistical power is low. Task-based fMRI has not seen widespread clinical use (outside of mapping for surgical planning) because of limitations on the number of tasks, difficulties associated with presenting tasks and/or training subjects on the task in a busy clinical MR center, and the lack of whole-brain assessment from such studies.

Functional connectivity data is different from task-based fMRI data in that it does not provide information as to which areas of the brain are differentially involved in the execution of a specific task, but instead provides a more basic measure reflecting how different brain areas are functionally connected to one another. As such, functional connectivity studies are potentially more appropriate for whole-brain surveys of functional abnormalities such as the clinical challenge in epilepsy of identifying seizure-generating tissue elements or networks. The hope is that examining the network properties of the seizure-generating tissue will lead to a better understanding of the impact of epilepsy on the functional organization of the brain, assist in understanding comorbidities, and facilitate surgical planning, which in turn could lead to improved surgical outcome through identification and resection of the critical node(s) in the seizure-generating networks(s). Connectivity data could, for example, be used to identify brain areas to target with invasive intracranial recording electrodes. Resting-state fMRI is not limited to identifying tissue to be resected but also has the potential to predict cognitive change following resection if the resection plan involves specific nodes in functional networks. Research in all of these areas is ongoing and these topics are discussed in further detail in the sections that follow.

Functional connectivity mapping was first described by Biswal et al. (1995) in a seminal paper, revealing correlations between brain regions that are synchronized in time, through spontaneous fluctuations of activity, while the brain regions themselves may be quite distant. Brain regions with high resting-state temporal correlations are thought to be involved in the same network and the more well-formed the network, the stronger the connectivity. Spontaneous fluctuations lead to changes in the BOLD signal and most of the work to date has focused on very low-frequency (<0.1 Hz) fluctuations.

Spontaneous fluctuations have also been studied with both surface EEF and intracranial recordings (Towle et al., 1999; Andrzejak et al., 2001, 2006; Schevon et al., 2007; Ortega et al., 2008a,b; Lehnertz et al., 2011; Palmigiano et al., 2012) and local differences in the connectivity between nodes in the seizure focus area have been observed. For a review discussing both increases and decreases in synchronization and their role in epilepsy please see Jiruska et al. (2013). The EEG work, it should be noted, has primarily focused on higher frequency fluctuations in the 1–40 Hz range although several groups now are beginning to target much lower frequency oscillations.

After the initial paper by Biswal et al. (1995), much effort was focused over the next decade on verifying that the correlations observed between brain regions indeed reflect true functional connections and not simply noise correlations due to physiological fluctuations. Without doubt, care must be taken to minimize the effects of physiologic noise as there are clear correlations associated simply with respiratory and cardiac signal fluctuations; however, the current consensus is that meaningful functional connections are found in continuously recorded BOLD fMRI data. Since about 2005, the field of functional connectivity mapping has been expanding rapidly through novel approaches to analysis, noise removal methodology, and applications to clinical populations and basic neuroscience problems. In particular, the field is expanding to more in-depth analyses that move beyond simply examining correlations between two or a small handful of brain regions to capturing network information from a large array of nodes across the brain.

For most functional connectivity studies, BOLD fMRI data is collected with the subject in the resting state with eyes either open or closed, and the patient is not required to perform a task. It is also possible to obtain connectivity data in the presence of a task and/or after brain-state manipulations, but it should be noted that the connectivity patterns can be slightly modified by task or brain state. Functional connectivity patterns have been shown to be sensitive to brain state as well as behavioral variables (Hampson et al., 2006a,b; Johnson et al., 2006; Rogers et al., 2010; Bonelli et al., 2012; Cole et al., 2012), and to vary with development (Fair et al., 2008; Schafer et al., 2009; Myers et al., 2010) and age (Dosenbach et al., 2010; Hampson et al., 2012).

Connectivity changes have also been reported in several clinical populations (Quigley et al., 2001; Lowe et al., 2002; Irwin et al., 2004; Saini et al., 2004; Haas et al., 2006; Waites et al., 2006; Hoffman et al., 2007; Schafer et al., 2009; Wang et al., 2009; Freilich and Gaillard, 2010; Myers et al., 2010; Bai et al., 2011; Killory et al., 2011; Zhang et al., 2011a,b; Bagshaw and Cavanna, 2012; de Groot et al., 2012) and there are now more than 100 publications related to connectivity measures in epilepsy patients. There is evidence that correlations between time-varying BOLD signals reflect intrinsic functional connections in that they are present when subjects are both awake and under anesthesia (Vincent et al., 2007; Martuzzi et al., 2010, 2011) and they are highly reproducible (Shehzad et al., 2009). Overall, resting-state functional connectivity mapping has significant potential to reveal the functional organization of the brain and how it may be altered in different diseases or disorders.

This review begins with an introduction to resting-state functional connectivity, including basic data collection and processing steps, the type of information that can be obtained, and various means of application. The particulars associated with resting-state methodology as applied in epilepsy are considered including strategies for voxel-based and region-of-interest (ROI) based analyses. Issues associated with selecting a connectivity threshold or avoiding such thresholds, ROIs, and the sensitivity of the method to choice of ROI are considered in addition to the emerging field of connectivity-based parcelation for identifying minimal functional subunits for nodal analysis. Finally we provide a number of early clinical results reflecting the potential of functional connectivity data to contribute to the clinical management of epilepsy.

## The Basics of Functional Connectivity Data Collection

Resting-state data is typically collected using a gradient-echo echo planar imaging (EPI) pulse sequence that provides whole-brain coverage with a temporal resolution of 3 s or less at a field strength of 3 T. Current state-of-the-art EPI involves the use of a multi-band/multi-plexed EPI sequences (Feinberg et al., 2010) that can provide whole-brain coverage with 2 mm^3^ voxel dimensions and a repetition time (TR) of less than 1 s. Short TR in fMRI generally provides better statistical power (Constable and Spencer, 2001) and eases removal of physiological noise in connectivity data (Lowe et al., 1998). A minimum of approximately 5 min of such data is required to obtain connectivity maps with reasonable signal-to-noise ratios (SNRs). Such an acquisition easily fits within the constraints of a clinical MRI study, although in general acquiring more data is advantageous.

Initial post-processing steps involve motion correcting the data (to ensure all volume acquisitions are aligned through time), and removing time-course signals that may be of no interest including the mean global signal through time, components associated with cerebrospinal fluid and white-matter signals, and the variables describing subject motion (the three translation and three rotation directions). Temporal drift terms are often regressed from the data and a low pass filter (<0.1 Hz) is applied.

The extent to which regressing the temporal fluctuations associated with motion is effective has been a topic of much discussion in the literature (Weissenbacher et al., 2009) and there is evidence for residual motion effects after correction (Power et al., 2012; Satterthwaite et al., 2012, 2013; Van Dijk et al., 2012). The issue of whether or not to remove the global signal mean also remains a hot topic as this can lead to the introduction of negative correlations in the data, making interpretation difficult (Fox et al., 2009; Murphy et al., 2009; Hampson et al., 2010).

Following the steps in the chosen preprocessing pipeline, functional connectivity is measured as the temporal correlation between the signal from any pair of voxels or ROIs. The following sections discuss the pros and cons of each approach in detail.

## Connectivity Measures

Resting-state connectivity mapping has been used to examine functional connections between cortical regions since the first presentation of the method by Biswal et al. (1995). In the sections that follow, we discuss how connectivity analyses can be applied to epilepsy for examining alterations in normal brain networks as a function of disease, or as a function of surgical intervention, as well as how connectivity mapping can be used to detect segments of tissue with abnormal functional connectivity with the goal of identifying the seizure-generating foci or network.

## ROI-to-Whole-Brain

**Figure 1:**
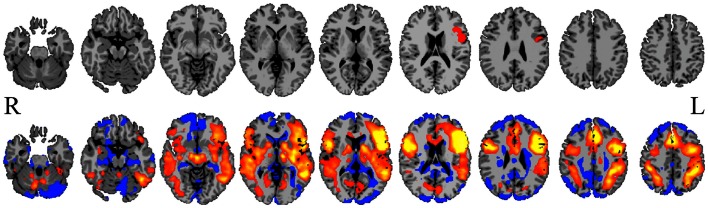
**Seed-to-whole-brain connectivity mapping**. A reference BOLD signal time-course from a seed in Broca’s area (top row) is correlated with the BOLD time-course for all gray-matter voxels in the image, revealing areas to which the seed is functionally connected (bottom row; hot colors = strong positive correlation, cool colors = weak or negative correlation). Such seed-to-whole-brain connectivity maps may be examined for a single epilepsy patient relative to a group of healthy control subjects, or for a group of patients with a similar pathology to a group of healthy control subjects, or correlated with behavioral measures. This approach and the results it produces are highly dependent upon how the initial seed region is chosen.

Defining a ROI and performing ROI-to-whole-brain connectivity analysis is probably the most common approach to examining connectivity in the brain; this was the original method introduced by Biswal et al. (1995). Such an approach is motivated when an investigator is interested in a particular brain region (the seed ROI) and wishes to examine what other brain regions the seed is connected to, as well as how such connections vary between healthy controls and a patient group, or pre- and post-surgical intervention (see Figure 1 for an example). In epilepsy this approach has been used to examine changes in language networks before and after anterior temporal lobe resection (Bonelli et al., 2012). In another study, Pereira et al. (2010) examined connectivity between the left and right hippocampi in mesial temporal sclerosis patients and found that relative to control subjects, patients with left hippocampal sclerosis showed a larger decrease in functional connectivity than patients with right hippocampal sclerosis in subjects with left-hemispheric language dominance. Morgan et al. (2012) performed a similar study using each of the left and right hippocampi as seeds for a seed-to-whole-brain analysis. This work revealed that the connectivity between the right hippocampus and the ventral lateral nucleus of the right thalamus could distinguish between seizure-free patients with left temporal lobe epilepsy (TLE) and right TLE patients and that in general, connectivity was greater in the seizure-free patient group with left TLE compared to the healthy controls.

In another study of the medial temporal lobe, Pittau et al. (2012) selected four manually drawn ROIs and examined differences in connectivity between 23 patients with mesial TLE and compared these to 23 age- and gender-matched controls in an ROI-to-whole-brain analysis. They found that patients with right MTLE had decreased connectivity between right amygdala and right hippocampus and the brain areas associated with the default mode network, some prefrontal regions, and contralateral mesial temporal structures. The left MTLE patients showed decreased connectivity between the left amygdala and left hippocampus to the default mode network, contralateral hippocampus, and bilateral limbic prefrontal regions.

Moving outside the temporal lobe, a study by Killory et al. (2011) performed ROI-to-whole-brain analysis using eight ROIs: three defined used a functional task-based paradigm and another five spherical ROIs based on previous literature. They then compared the resulting maps from patients with childhood absence epilepsy to those of control subjects. An overall decrease in connectivity in the attention nodes was observed in the absence patients, who demonstrated impaired connectivity in the insula/frontal operculum and medial frontal nodes relative to healthy control subjects.

In a study that used simultaneous EEG/fMRI to define the initial seed regions, Negishi et al. (2011) compared connectivity to the seed within the same hemisphere and the contralateral hemisphere to derive a connectivity laterality index. Results showed decreased laterality of functional connectivity in the patients that were seizure-free after surgery compared to those that had recurrent seizures after surgery.

## ROI-to-ROI

Another approach to connectivity analysis is to define a set of ROIs and to measure functional connectivity between all possible pairs of ROIs. This has been applied in numerous studies of epilepsy and some of these are summarized below. Once multiple ROIs are considered, network properties for these ROIs can be measured as discussed in the section on network theoretic measures below.

In a study by Bettus et al. (2010), the investigators chose five ROIs in each hemisphere – primarily in medial temporal lobe regions, defined using the Pick atlas tool in SPM (Maldjian et al., 2003) – and examined connectivity between each pair of ROIs and compared this to data from 36 control subjects. They reported decreased connectivity in mesial TLE patients relative to control subjects, with the largest decreases on the ipsilateral side and some increased connectivity on the contralateral side. In a similar study, Morgan et al. (2011) defined hippocampal ROIs using both structural and functional information and revealed a relationship between functional connectivity and causal influence of the left and right hippocampi that varied with the duration of disease in a group of 19 mesial TLE patients. Looking at a broader network of nodes in a subsequent paper, Morgan et al. (2012) compared pre-operative fMRI connectivity data in two groups of patients who were postoperatively sorted into seizure-free and those with recurring seizures. They showed that the connectivity between the right hippocampus and the ventral lateral nucleus of the right thalamus could distinguish between seizure-free patients with left TLE from those with right TLE with high sensitivity and specificity. The patients with recurring seizures after surgery generally exhibited different connectivity values in this network from those that were seizure-free.

In a study of 11 children with intractable epilepsy, Widjaja et al. (2013) used independent components analysis to identify the default mode network and then calculated the functional connectivity between different pairs of ROIs in this network. They found reduced connectivity in the default mode network in children with medically refractory epilepsy.

Using ROI-to-ROI connectivity analyses with a number of predefined ROIs, Bai et al. (2011) investigated functional connectivity in a group of childhood absence epilepsy patients. In a unique twist on the ROI approach, they also examined ROI homologs in a cross-hemispheric study where the ROIs in this case were individual voxels. The primary finding was increased interhemispheric connectivity in the lateral orbitofrontal cortex in the patient group relative to the healthy control subjects.

A pattern classification approach was applied to another ROI-to-ROI study in epilepsy by Zhang et al. (2011a). This work examined connectivity patterns between nodes in both hemispheres and found, much like the Negishi et al. (2011) study described in the previous section, that asymmetry in functional connectivity could correctly distinguish epilepsy patients from healthy controls with 82.5% specificity and 85% sensitivity.

## Network Theoretic Measures

While ROI-to-whole-brain and ROI-to-ROI analyses can reveal much about functional connectivity, such approaches do not take the next step of considering nodes in the context of a functional network and the properties of that network as a whole. Recently there has been an explosion of interest in applying network theory (see Achard et al., 2006; Bullmore and Sporns, 2009; Bressler and Menon, 2010; Hagmann et al., 2010; Rubinov and Sporns, 2010) to the analysis of functional connectivity data in order to characterize brain connections at both the nodal and network level. This allows for the observation of the networks associated with specific brain functions and generally moves fMRI from identification of individual nodes to systems involved in the execution of a task. Such theory, when applied to resting-state fMRI data, can characterize the topology of normal networks in the brain and, by extension, identify abnormal patterns of connectivity.

Network theory measures have been applied in epilepsy to EEG or intracranial EEG data (Ponten et al., 2009, 2010; van Dellen et al., 2009; Varotto et al., 2012), MEG data (Chavez et al., 2010), and cortical thickness correlations (Bernhardt et al., 2011), but to our knowledge only one published study to date has specifically examined network properties using fMRI data (Zhang et al., 2011a; although in the voxel-based approaches section below we include a small study by Stufflebeam that uses the graph-theory measure of degree to attempt to identify epileptogenic tissue). In this work, a total of 36 ROIs were defined using both functional (language and motor regions) and anatomic definitions [Brodmann’s (1909) areas]. Five network measures – degree, strength, clustering coefficient, closeness, and betweenness centrality (Wasserman and Faust, 1994) – were calculated for these nodes in individual subjects as well as for input into a classification strategy to determine if such measures could distinguish medial TLE patients from healthy control subjects. The results confirmed that network measures such as these can indeed aid in classifying epilepsy patients and that the epilepsy process is associated with changes in network-level functional brain organization.

Taken together, independent of whether the analysis is ROI-to-whole-brain, ROI-to-ROI, or at the level of network properties, these studies suggest that functional connectivity has potential for identifying disrupted circuitry as a function of disease and, perhaps more importantly, predicting outcomes from surgical intervention. In the sections that follow we outline some important issues encountered in these studies related to defining ROIs and determining thresholds for connectivity.

## ROI-Based Approaches and the Problem of ROI Definition

As described above, many connectivity analyses require the *a priori* definition of at least one ROI. While not often highlighted in previous publications, the choice of the seed ROI(s) and how the exact boundaries of that ROI are defined is critical. If an ROI contains multiple time-courses then the average time-course from the ROI may not properly represent any of the time-courses within an ROI and the results may be completely erroneous. Further, varying the spatial definition of the seed can substantial changes in results. This is easily highlighted by observing that in a typical ROI-to-whole-brain connectivity map (e.g., see Figure 1), there are often sharp transitions from positive to negative correlations; hence, moving the seed can result in a very different map.

As is evident in the papers already discussed, numerous approaches to defining ROIs have been used to date. Task-based fMRI has been used to define specific functional circuits within which connectivity can be analyzed (Frings et al., 2009; Bonelli et al., 2012). This approach, however, suffers from the limitations of task-based fMRI studies in general in that only a very limited number of ROIs are activated by a task and thus whole-brain assessment of connectivity is not possible using such definitions. Another approach has been to use independent component analyses (ICA) (McKeown et al., 1998) to delineate brain regions (Luo et al., 2012; Mankinen et al., 2012) but these have typically identified only a very limited number of networks – often, for example, fewer than 10.

Anatomic ROI definitions have also been used extensively (Crespo-Facorro et al., 1999; Tzourio-Mazoyer et al., 2002; Makris et al., 2005; Shattuck et al., 2008; Zhang et al., 2011a). Such definitions are ideal in structures that are well-defined anatomically (such as the hippocampus) but are difficult in areas such as the frontal and parietal cortices, and therefore the risk of mixing temporal signals into heterogeneous ROIs in these regions is high. Many investigators have used small spherical ROI placements (Shehzad et al., 2009; Bai et al., 2011; Bettus et al., 2011; Killory et al., 2011; Koyama et al., 2011); in this case the risk of mixing different functional time-courses decreases with the size of the defined sphere but is not eliminated. Many investigators have arbitrarily parcelated the cortex into anywhere from 100 to 1000 nodes, but again, with such an approach, the node definitions may not necessarily reflect true functional boundaries.

**Figure 2:**
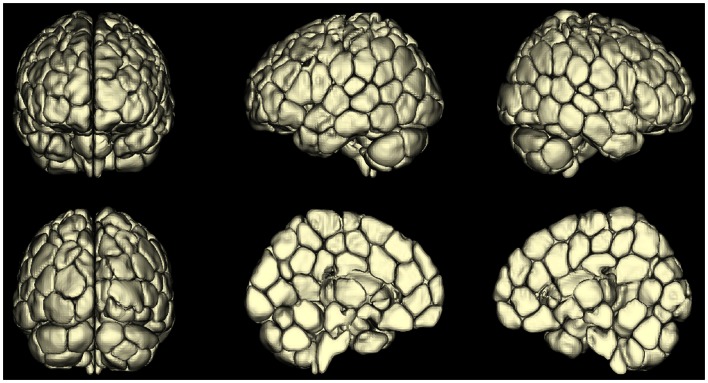
**A map of reproducible (across 79 subjects) functional subunits identified using resting-state connectivity data**. These functional subunits are ideal nodes for connectivity analyses as they have highly uniform time-courses for each voxel within a given node by definition. This parcelation approach (Shen et al., 2010, 2013) provides a solution to the ROI definition problem in connectivity or network analysis of the brain.

An emerging area of investigation involves performing whole-brain parcelation based on the time-courses themselves (van den Heuvel et al., 2008; Shen et al., 2010, 2013; Craddock et al., 2012). This approach appears very promising because it can provide minimal functional subunits with uniform time-courses within each unit. An example obtained using the approach of Shen et al. (2013) is shown in Figure 2. Both Shen et al. (2013) and Craddock et al. (2012) have shown that ROIs extracted from these parcelations had higher functional homogeneity than anatomically defined ROIs and thus were more relevant for fMRI connectivity analyses. This parcelation approach using connectivity data itself appears to solve the problem of providing whole-brain ROI definitions for meaningful connectivity analysis. The next problem is how to apply such an approach to a patient population or to a group of patients. For example, if one generates a parcelation from healthy control subjects to investigate differences in network properties between control subjects and epilepsy patients, any results could be interpreted as due to actual differences in network properties, or a mismatch in the ideal functional boundaries for the parcelation nodes that come from imposing a control-derived parcelation on patients. This latter question can be addressed by directly comparing a parcelation derived from the patient or patient group to the parcelation derived from the healthy control subjects.

Thus, while it has been difficult to define ROIs for functional connectivity analysis, these new parcelation approaches appear to have solved this problem. Another approach, however, is to avoid the ROI problem completely by moving to voxel-level connectivity analysis, in which each voxel in the gray matter is treated as an individual node and summary statistics on the network or connectivity properties of each voxel are obtained. This approach is described in the next section.

## Voxel-Based Connectivity Analysis

**Figure 3:**
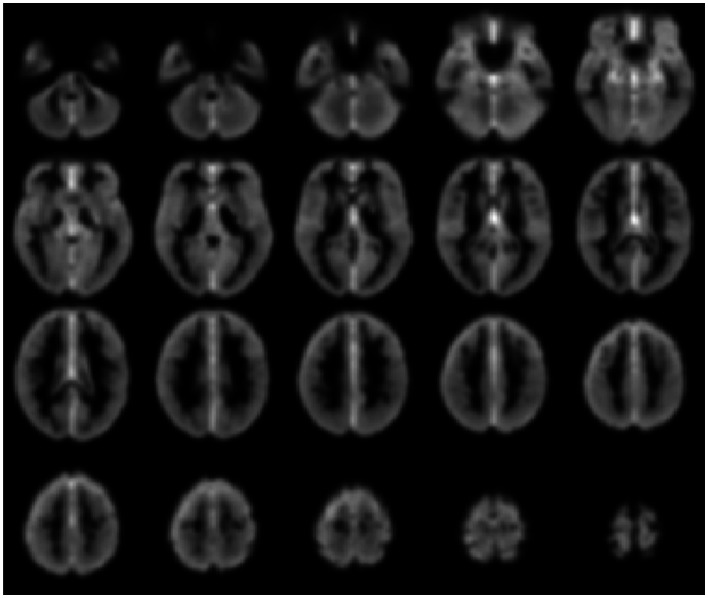
**Gray-scale short axis MR images (from *n* = 42 healthy control subjects) with contrast reflecting the functional connectivity of each voxel as measured by the network measure of degree (brighter colors indicate higher degree)**. Such maps can be obtained for individual patients and compared to control-group data to isolate tissue elements with abnormal functional connectivity.

Voxel-level network analyses have been developed that can provide insight into the functional connectivity of individual tissue elements, and a number of important studies have been published using such approaches (Buckner et al., 2009; Martuzzi et al., 2011; Stufflebeam et al., 2011; Scheinost et al., 2012). Voxel-level analyses have the benefit of not requiring *a priori* definition of a ROI, instead treating each voxel as a node in a network analysis. In this approach a network measure such as *degree* can be calculated for each voxel. (*Degree* is the number of connections to a voxel above some arbitrary correlation threshold, i.e., *r* > *t*, where *t* = 0.25). Such a degree map, as shown in Figure 3 below, provides then for the first time a gray-scale contrast reflecting the functional connectivity of each tissue element and is a potentially powerful approach to identifying regions that have abnormal functional connectivity on a whole-brain level. Such degree maps can also be used to define nodes for further ROI-to-ROI network analyses and/or to compare individual patients with control-group data.

Such a voxel-based approach has been used to study brain changes in Alzheimer’s disease (Buckner et al., 2009), to examine the effects of anesthetics on the human brain (Martuzzi et al., 2011) and to identify epileptogenic tissue in epilepsy (Stufflebeam et al., 2011).

**Figure 4:**
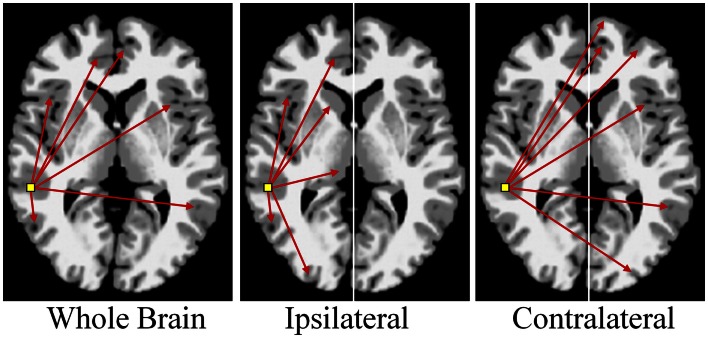
**Calculation of functional connectivity maps from resting-state fMRI data**. The network measure of degree, reflecting functional connectivity for a single voxel, is calculated by counting the number of connections to that voxel above a correlation threshold across the whole-brain (bilateral; left panel), within the same hemisphere (ipsilateral; middle panel), or to the opposite hemisphere (contralateral; right panel).

There are multiple approaches to calculating degree; three simple schematics are shown in Figure 4 to illustrate the different approaches. In these examples the voxel-based degree measure can be calculated for connections encompassing the entire brain (whole-brain connectivity), within the same hemisphere as the voxel (ipsilateral connectivity) or spanning connections to the other hemisphere (contralateral connectivity). In each case the calculation is performed for each gray-matter voxel in the brain and the intensity of each voxel then reflects its number of connections above a predetermined correlation threshold.

**Figure 5:**
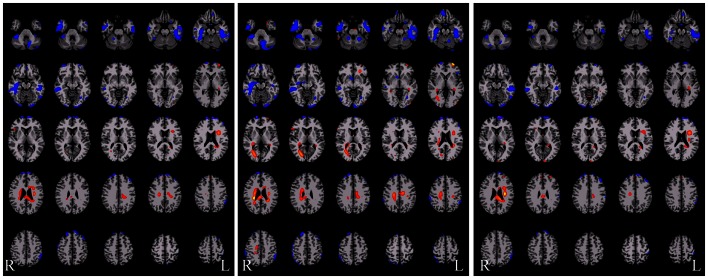
**Functional connectivity difference maps for a single epilepsy patient versus a group of healthy control subjects (red = connectivity increased in patient relative to controls; blue = connectivity decreased in patient relative to controls)**. Some of these regions are consistent with the seizure onset zones but clearly a large number of regions show altered connectivity. There are subtle differences between these three measures and more work is needed to determine the sources of these differences and the interactions with different pathological variants.

An example of such an approach applied in epilepsy is shown in Figure 5 below where the three *degree* measures are shown for a single patient relative to 20 healthy control subjects. The idea behind this approach is that seizure-generating tissue may have altered functional connectivity either because of the epilepsy processes themselves or because the tissue is not functioning normally. Generally, in all three measures, we observe widespread decreases along with some increases in functional connectivity for the patient relative to the controls. Much needs to be learned about this approach, however, as there are subtle differences in the ipsilateral and contralateral measures that may provide relevant information for specific pathologies. In the case shown in Figure 5 the patient had suspected right medial TLE and indeed there is a large region of decreased functional connectivity in the right temporal lobe relative to healthy control subjects across all three measures. However, there are many other regions that also show differences in functional connectivity relative to control subjects and it is currently an open question whether these differences reflect part of the epilepsy network, brain reorganization of functional subunits as a result of having epilepsy, or some other neurophysiological mechanism.

**Figure 6:**
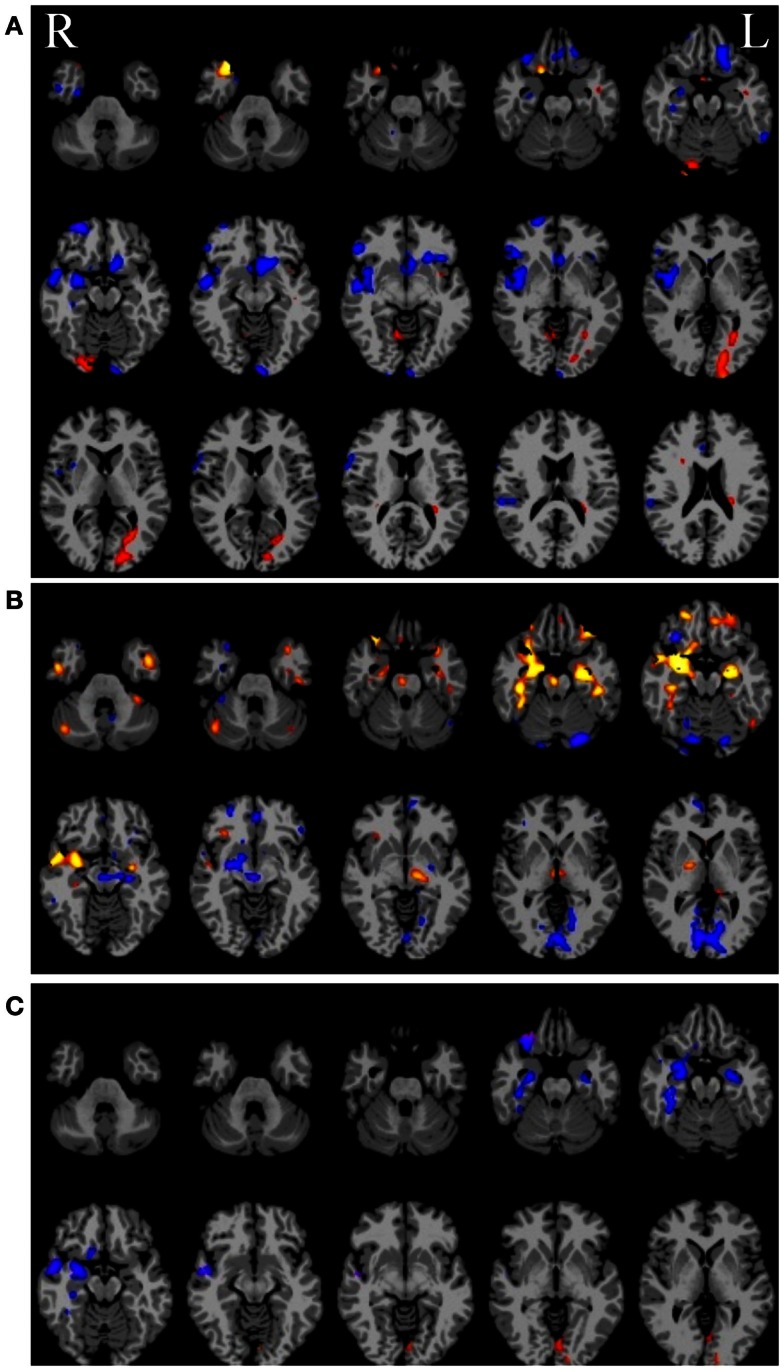
**(A)** Ipsilateral connectivity for a single patient versus a group of healthy control subjects showing multiple regions with altered connectivity (warm colors = higher connectivity for patient; cool colors = higher connectivity for controls). **(B)** In an individual analysis of the patient data, selecting the right hippocampus, which was the onset zone suspected from clinical data, as a seed region reveals a network of nodes with high vs. low connectivity to the right hippocampus. In **(C)** the intersection of the maps in **(A)** and **(B)** highlights regions that have abnormal connectivity compared to controls AND are part of the same network involving the right hippocampus.

Given some clinical consensus via more conventional measures (surface and/or video EEG, other clinical data, and/or invasive recordings) there is often some indication that the patient has foci in a particular region or lobe. In the next example, shown in Figure 6 below, the right hippocampus was suspected to be part of the seizure-generating network and indeed the ipsilateral degree measure showed decreased functional connectivity in this region for the patient (see Figure 6A). (As an aside, we note that the ipsilateral degree measure is perhaps the most straightforward and easiest to interpret, whereas the whole-brain and contralateral measures are more difficult to understand in terms of the lateralization of the source of the problem.) As in the previous example, many other regions also show alterations in connectivity and some of these may be part of the seizure-generating network. Still, with *a priori* clinical information about the suspected site of seizure onset, it is reasonable to use the right hippocampus as a seed ROI for an ROI-to-whole-brain analysis. The nodes connected to this right hippocampal region are shown in Figure 6B with hot colors reflecting strong connectivity to the seed region. Combining the ipsilateral connectivity map in Figure 6A with the map showing connections to the suspected focal region in Figure 6B using a logical AND operation, yields a much more circumscribed map Figure 6C, which highlights regions that have both altered functional connectivity and are part of the same network. (The most superior slices in the brain are not shown in the bottom two panels as there are no regions in these slices that satisfy both constraints.)

Yet, despite their potential, voxel-based analyses of connectivity have not seen widespread application primarily for two reasons: (1) a lack of sensitivity, and (2) vulnerability to threshold effects.

The first problem is partially solved by higher-field magnets of 3 T or more as well as the more recent move to ultrafast pulse sequences such as the multi-band/multi-plexed sequence developed by Feinberg et al. (2010). In addition, sensitivity can be increased further by examining the distribution of degree values across the entire range of thresholds; this also addresses the second problem, as described in the next section.

The second problem, the need for an arbitrary threshold to calculate the degree measure for each voxel, is unique to the application of network theory measures to fMRI data. In most network theory applications, such as tracking the transmission of a disease or analyzing friend links on Facebook, the decision tree is binary – either the disease is transferred or it is not; either two people are friends or they are not. However, in the case of fMRI connectivity, since connectivity is measured as a correlation, values may span a continuum from −1 to 1. Thus, an arbitrary threshold is typically invoked to decide if two regions are connected or not. In general there is no principled way to choose this threshold and the results can change dramatically at different thresholds, which is a major issue for applying network or graph-theory measures (Rubinov and Sporns, 2010) to fMRI data. To get around this arbitrary threshold choice, Scheinost et al. (2012) presented a new approach that examines the entire connectivity distribution curve across all correlation thresholds from 0 to 1. (The absolute value of the correlations can be taken, therefore taking negative correlations into account, or the positive and negative correlations can be treated in separate analyses). This new approach, referred to as the intrinsic connectivity distribution, is described in the section that follows.

## Intrinsic Connectivity Distribution Analysis

**Figure 7:**
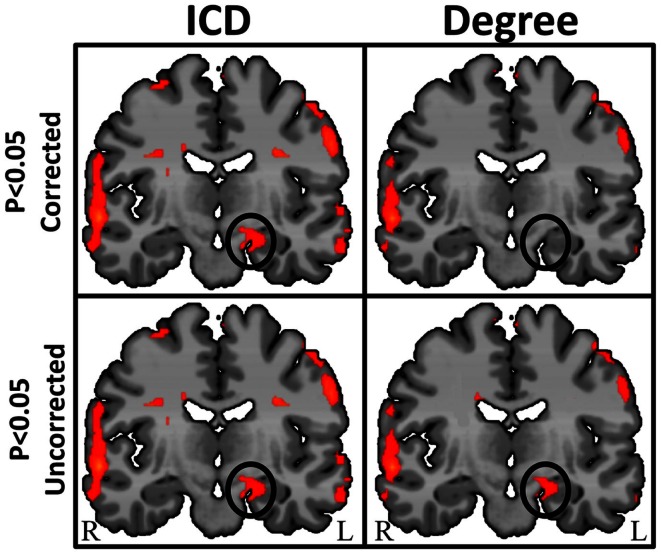
**Regions of high intrinsic connectivity for left temporal lobe epilepsy (LTE) patients (*n* = 10) shown at different statistical levels**. At *p* < 0.05 corrected (top row) the ICD map (left) highlights significant patterns of increased connectivity in the left hippocampus whereas the degree map (right) does not show significantly different connectivity in this region. At a less stringent statistical threshold (bottom row), the degree map shows the same areas at *p* < 0.05 uncorrected (right) as the ICD maps (left) at either statistical threshold. This suggests that intrinsic connectivity is capable of detecting abnormal connectivity in regions of epileptogenic tissue and that the ICD approach yields higher sensitivity and reproducibility.

By characterizing the entire *degree* curve for any of the three voxel-based connectivity metrics described above (whole-brain, ipsilateral, and contralateral), the need for a specific but arbitrary threshold is eliminated, providing both a more consistent measure of functional connectivity and a more sensitive measure of individual differences. Summarizing the entire degree distribution curve captures information about all connections, weak to strong. This parametric estimation approach therefore enables the interpretation of any differences (between an epilepsy patient and a control group, for example) to take the form of “more stronger connections,” “more weaker connections,” or “more even spread of connections.” An example in a group of 10 TLE patients comparing degree at a single threshold (*r* > 0.25) and ICD is shown in Figure 7.

The ICD approach improves on the earlier voxel-based degree measure through increased sensitivity as well as through more stable interpretation of the results. To date no comprehensive study has been published using these emerging methods, but our lab as well as others are currently applying this to the problem of localizing seizure foci or networks in epilepsy and no doubt more studies will appear shortly.

## Summary

In summary, resting-state functional connectivity as measured by BOLD fMRI reflects intrinsic connections in the brain, providing insight into how the brain is wired and how such wiring may be altered in disease or through surgical intervention. Like task-based fMRI, functional connectivity measures can be altered by task, drug or brain state, but unlike task-based fMRI, which reflects small changes in activity superimposed upon a very high baseline activity level, functional connectivity measures intrinsic functional connections that exist even in the presence of deep anesthesia.

The measures of intrinsic connectivity as described above, particularly the voxel-based degree measure, provide for the first time a contrast mechanism in MRI based on function rather than anatomy. Since this approach requires no task, the data is acquired much in the same way that anatomic MRI data is acquired and thus these measures can be easily incorporated into clinical diagnostic radiology departments.

Furthermore, while task-based fMRI provides functional information on only a very limited number of cortical regions (i.e., those few regions differentially activated by the task), the voxel-based connectivity measures described above provide functional information on the whole-brain without the need for a task. This makes it ideal for investigating the effects of pharmacological agents and the impact of diseases and specific pathologies on the brain without the need for *a priori* knowledge or selection of ROI. The use of functional connectivity methods in epilepsy will undoubtedly increase as we learn more about the functional changes that occur with epilepsy and/or as a function of surgical intervention.

## Conflict of Interest Statement

The authors declare that the research was conducted in the absence of any commercial or financial relationships that could be construed as a potential conflict of interest.

## References

[B1] Achard S. Salvador R. Whitcher B. Suckling J. Bullmore E. (2006). A resilient, low frequency, small-world human brain functional network with highly connected association cortical hubs. Neuroscience 26, 63–72 10.1523/JNEUROSCI.3874-05.2006 16399673 PMC6674299

[B2] Andrzejak R. G. Mormann F. Widman G. Kreuz T. Elger C. E. Lehnertz K. (2006). Improved spatial characterization of the epileptic brain by focusing on nonlinearity. Epilepsy Res. 69, 30–44 10.1016/j.eplepsyres.2005.12.004 16503398

[B3] Andrzejak R. G. Widman G. Lehnertz K. Rieke C. David P. Elger C. E. (2001). The epileptic process as nonlinear deterministic dynamics in a stochastic environment: an evaluation on mesial temporal lobe epilepsy. Epilepsy Res. 44, 129–140 10.1016/S0920-1211(01)00195-4 11325569

[B4] Bagshaw A. P. Cavanna A. E. (2012). Resting state networks in paroxysmal disorders of consciousness. Epilepsy Behav. 26, 290–294 10.1016/j.yebeh.2012.09.020 23089152

[B5] Bai X. Guo J. Killory B. Vestal M. Berman R. Negishi M. (2011). Resting functional connectivity between the hemispheres in childhood absence epilepsy. Neurology 76, 1960–1967 10.1212/WNL.0b013e31821e54de 21646622 PMC3109878

[B6] Bernhardt B. C. Chen Z. he Y. Evans A. C. Bernasconi N. (2011). Graph-theoretical analysis reveals disrupted small-world organization of cortical thickness correlation networks in temporal lobe epilepsy. Cereb. Cortex 21, 2147–2157 10.1093/cercor/bhq291 21330467

[B7] Bettus G. Bartolomei F. Confort-Gouny S. Guedj E. Chauvel P. Cozzone P. J. (2010). Role of resting state functional connectivity MRI in presurgical investigation of mesial temporal lobe epilepsy. J. Neurol. Neurosurg. Psychiatr. 81, 1147–1154 10.1136/jnnp.2009.191460 20547611

[B8] Bettus G. Ranjeva J. P. Wendling F. Bénar C. G. Confort-Gouny S. Régis J. (2011). Interictal functional connectivity of human epileptic networks assessed by intracerebral EEG and BOLD signal fluctuations. PLoS ONE 6:e20071 10.1371/journal.pone.0020071 21625517 PMC3098283

[B9] Biswal B. B. Yetkin F. Z. Haughton V. M. Hyde J. S. (1995). Functional connectivity in the motor cortex of the resting human brain using echo-planar MRI. Magn. Reson. Med. 34, 537–541 10.1002/mrm.1910340409 8524021

[B10] Bonelli S. B. Thompson P. J. Yogarajah M. Vollmar C. Powell R. H. Symms M. R. (2012). Imaging language networks before and after anterior temporal lobe resection: results of a longitudinal fMRI study. Epilepsia 53, 639–650 10.1111/j.1528-1167.2012.03433.x 22429073 PMC4471632

[B11] Bressler S. L. Menon V. (2010). Large-scale brain networks in cognition: emerging methods and principles. Trends Cogn. Sci. (Regul. Ed.) 14, 277–290 10.1016/j.tics.2010.04.004 20493761

[B12] Brodmann K. (1909). Vergleichende Lokalisationslehre der Grosshirnrinde. Barth: Verlag

[B13] Buckner R. L. Sepulcre J. Talukdar T. Krienen F. M. Liu H. Hedden T. (2009). Cortical hubs revealed by intrinsic functional connectivity: mapping, assessment of stability, and relation to Alzheimer’s disease. Neuroscience 29, 1880–1893 10.1523/JNEUROSCI.5062-08.2009 PMC2750039 19211893

[B14] Bullmore E. Sporns O. (2009). Complex brain networks: graph theoretical analysis of structural and functional systems. Nature 10, 186–198 10.1038/nrn2575 19190637

[B15] Chavez M. Valencia M. Navarro V. Latora V. Martinerie J. (2010). Functional modularity of background activities in normal and epileptic brain networks. Phys. Rev. Lett. 104, 118701 10.1103/PhysRevLett.104.118701 20366507

[B16] Cole M. W. Yarkoni T. Repovs G. Anticevic A. Braver T. S. (2012). Global connectivity of prefrontal cortex predicts cognitive control and intelligence. J. Neurosci. 32, 8988–8999 10.1523/JNEUROSCI.0536-12.2012 22745498 PMC3392686

[B17] Constable R. T. Spencer D. D. (2001). Repetition time in echo planar functional MR imaging. Magn. Reson. Med. 46, 748–755 10.1002/mrm.1253 11590651

[B18] Craddock R. C. James G. A. Holtzheimer P. E. Hu X. P. Mayberg H. S. (2012). A whole-brain fMRI atlas generated via spatially constrained spectral clustering. Hum. Brain Mapp. 33, 1914–1928 10.1002/hbm.21333 21769991 PMC3838923

[B19] Crespo-Facorro B. Cabranes J. A. López-Ibor Alcocer M. I. Payá B. Fernández Pérez C. Encinas M. (1999). Regional cerebral blood flow in obsessive-compulsive patients with and without a chronic tic disorder. A SPECT study. Eur. Arch. Psychiatry Clin. Neurosci. 249, 156–161 10.1007/s004060050081 10433130

[B20] de Groot M. Reijneveld J. C. Aronica E. Heimans J. J. (2012). Epilepsy in patients with a brain tumour: focal epilepsy requires focused treatment. Brain 135(Pt 4), 1002–1016 10.1093/brain/awr310 22171351

[B21] Dosenbach N. U. Nardos B. Cohen A. L. Fair D. A. Power J. D. Church J. A. (2010). Prediction of individual brain maturity using fMRI. Science 329, 1358–1361 10.1126/science.1194144 20829489 PMC3135376

[B22] Fair D. A. Cohen A. L. Dosenbach N. U. Church J. A. Miezin F. M. Barch D. M. (2008). The maturing architecture of the brain’s default network. Proc. Natl. Acad. Sci. U.S.A. 105, 4028–4032 10.1073/pnas.0800376105 18322013 PMC2268790

[B23] Feinberg D. A. Moeller S. Smith S. M. Auerbach E. Ramanna S. Gunther M. (2010). Multiplexed echo planar imaging for sub-second whole brain FMRI and fast diffusion imaging. PLoS ONE 5:e15710 10.1371/journal.pone.0015710 21187930 PMC3004955

[B24] Fox M. D. Zhang D. Snyder A. Z. Raichle M. E. (2009). The global signal and observed anticorrelated resting state brain networks. J. Neurophysiol. 101, 3270–3283 10.1152/jn.90777.2008 19339462 PMC2694109

[B25] Freilich E. R. Gaillard W. D. (2010). Utility of functional MRI in pediatric neurology. Curr. Neurol. Neurosci. Rep. 10, 40–46 10.1007/s11910-009-0077-7 20425225

[B26] Frings L. Schulze-Bonhage A. Spreer J. Wagner K. (2009). Remote effects of hippocampal damage on default network connectivity in the human brain. J. Neurol. 256, 2021–2029 10.1007/s00415-009-5233-0 19603243

[B27] Haas B. W. Omura K. Amin Z. Constable R. T. Canli T. (2006). Functional connectivity with the anterior cingulated is associated with extraversion during the emotional Stroop task. Soc. Neurosci. 1, 16–24 10.1080/17470910600650753 18633773

[B28] Hagmann P. Cammoun L. Gigandet X. Gerhard S. Grant P. E. Wedeen V. (2010). MR connectomics: principles and challenges. J. Neurosci. Methods 194, 34–45 10.1016/j.jneumeth.2010.01.014 20096730

[B29] Hampson M. Driesen N. Roth J. K. Gore J. C. Constable R. T. (2010). Functional connectivity between task-positive and task-negative brain areas and its relation to working memory performance. Magn. Reson. Imaging 28, 1051–1057 10.1016/j.mri.2010.03.021 20409665 PMC2936669

[B30] Hampson M. Tokoglu F. Shen X. Scheinost D. Papademetris X. Constable R. T. (2012). Intrinsic brain connectivity related to age in young and middle aged adults. PLoS ONE 7:e44067 10.1371/journal.pone.0044067 22984460 PMC3439483

[B31] Hampson M. Tokoglu F. Sun Z. Schafer R. J. Gore J. C. Constable R. T. (2006a). Connectivity-behavior analysis reveals functional connectivity between left BA39 and Broca’s area varies with reading ability. Neuroimage 31, 513–519 10.1016/j.neuroimage.2005.12.040 16497520

[B32] Hampson M. Driesen N. R. Skudlarski P. Gore J. C. Constable R. T. (2006b). Brain connectivity related to working memory performance. J. Neurosci. 26, 13338–13343 10.1523/JNEUROSCI.3408-06.2006 17182784 PMC2677699

[B33] Hoffman R. E. Hampson M. Wu K. Anderson A. Gore J. C. Buchanan R. J. (2007). Probing the pathophysiology of auditory hallucinations by combining functional magnetic resonance imaging and transcranial magnetic stimulation. Cereb. Cortex 17, 2733–2743 10.1093/cercor/bhl183 17298962 PMC2634833

[B34] Irwin W. Anderle M. J. Abercrombie H. C. Schafer S. M. Kalin N. H. Davidson R. J. (2004). Amygdalar interhemispheric functional connectivity differs between the non-depressed and the depressed human brain. Neuroimage 21, 467–686 10.1016/j.neuroimage.2003.09.057 14980569

[B35] Jiruska P. de Curtis M. Jefferys J. G. R. Schevon C. A. Schiff S. J. Schindler K. (2013). Synchronization and desynchronization in epilepsy: controversies and hypotheses. J. Physiol. (Lond.) 591, 787–797 10.1113/jphysiol.2012.239590 23184516 PMC3591697

[B36] Johnson M. K. Raye C. L. Mitchell K. J. Touryan S. R. Greene E. J. Nolen-Hoeksema S. (2006). Dissociating medial frontal and posterior cingulate activity during self-reflection. Soc. Cogn. Affect. Neurosci. 1, 56–64 10.1093/scan/nsl004 18574518 PMC2435374

[B37] Killory B. D. Bai X. Negishi M. Vega C. Spann M. N. Vestal M. (2011). Impaired attention and network connectivity in childhood absence epilepsy. Neuroimage 56, 2209–2217 10.1016/j.neuroimage.2011.03.036 21421063 PMC3105167

[B38] Koyama M. S. Di Martino A. Zuo X. N. Kelly C. Mennes M. Jutagir D. R. (2011). Resting-state functional connectivity indexes reading competence in children and adults. J. Neurosci. 31, 8617–8624 10.1523/JNEUROSCI.4865-10.2011 21653865 PMC3893355

[B39] Lehnertz K. Andrzejak R. G. Arnhold J. Kreuz T. Mormann F. Rieke C. (2011). Nonlinear EEG analysis in epilepsy: its possible to use for interictal focus localization, seizure anticipation, and prevention. J. Clin. Neurophysiol. 18, 209–222 11528294 10.1097/00004691-200105000-00002

[B40] Lowe M. J. Phillips M. D. Lurito J. T. Mattson D. Dzemidzic M. Mathews V. P. (2002). Multiple sclerosis: low frequency temporal blood oxygen level-dependent fluctuations indicate reduced functional connectivity – initial results. Radiology 224, 184–192 10.1148/radiol.2241011005 12091681

[B41] Lowe M. L. Mock B. J. Sorensen J. A. (1998). Functional connectivity in single and multi-slice echoplanar imaging using resting-state fluctuations. Neuroimage 7, 119–132 10.1006/nimg.1997.0315 9558644

[B42] Luo C. Qiu C. Guo Z. Fang J. Li Q. Lei X. (2012). Disrupted functional brain connectivity in partial epilepsy: a resting-state fMRI study. PLoS ONE 7:e28196 10.1371/journal.pone.0029446 22242146 PMC3252302

[B43] Makris N. Schlerf J. E. Hodge S. M. Haselgrove C. Albaugh M. D. Seidman L. J. (2005). MRI-based surface-assisted parcellation of human cerebellar cortex: an anatomically specified method with estimate of reliability. Neuroimage 25, 1146–1160 10.1016/j.neuroimage.2004.12.056 15850732

[B44] Maldjian J. A. Laurienti P. J. Kraft R. A. Burdette J. H. (2003). An automated method for neuroanatomic and cytoarchitectonic atlas-based interrogation of fMRI data sets. Neuroimage 19, 1233–1239 10.1016/S1053-8119(03)00169-1 12880848

[B45] Mankinen K. Jalovaara P. Paakki J.-J. Harila M. Rytky S. Tervonen O. (2012). Connectivity disruptions in resting-state functional brain networks in children with temporal lobe epilepsy. Epilepsy Res. 100, 168–178 10.1016/j.eplepsyres.2012.02.010 22418271

[B46] Martuzzi R. Ramani R. Qiu M. Rajeevan N. Constable R. T. (2010). Functional connectivity and alterations in baseline brain state in humans. Neuroimage 49, 823–834 10.1016/j.neuroimage.2009.07.028 19631277 PMC2764802

[B47] Martuzzi R. Ramani R. Qiu M. Shen X. Papademetris X. Constable R. T. (2011). A whole-brain voxel based measure of intrinsic connectivity contrast reveals local changes in tissue connectivity with anesthetic without a priori assumptions on thresholds or regions of interest. Neuroimage 58, 1044–1050 10.1016/j.neuroimage.2011.06.075 21763437 PMC3183817

[B48] McKeown M. J. Makeig S. Brown G. G. Jung T. P. Kindermann S. S. Bell A. J. (1998). Analysis of fMRI data by blind separation into independent spatial components. Hum. Brain Mapp. 6, 160–188 10.1002/(SICI)1097-0193(1998)6:5/6<368::AID-HBM7>3.3.CO;2-5 9673671 PMC6873377

[B49] Morgan V. L. Rogers B. P. Sonmezturk H. H. Gore J. C. Abou-Khalil B. (2011). Cross hippocampal influence in mesial temporal lobe epilepsy measured with high temporal resolution functional magnetic resonance imaging. Epilepsia 52, 1741–1749 10.1111/j.1528-1167.2011.03196.x 21801166 PMC4428312

[B50] Morgan V. L. Sonmezturk H. H. Gore J. C. Abou-Khalil B. (2012). Lateralization of temporal lobe epilepsy using resting functional magnetic resonance imaging connectivity of hippocampal networks. Epilepsia 53, 1628–1635 10.1111/j.1528-1167.2012.03590.x 22779926 PMC3436984

[B51] Murphy K. Birn R. M. Handwerker D. A. Jones T. B. Bandettini P. A. (2009). The impact of global signal regression on resting state correlations: are anti-correlated networks introduced? Neuroimage 44, 893–905 10.1016/j.neuroimage.2008.09.036 18976716 PMC2750906

[B52] Myers E. H. Hampson M. Vohr B. Lacadie C. Frost S. J. Pugh K. R. (2010). Functional connectivity to a right hemisphere language center in prematurely born adolescents. Neuroimage 51, 1445–1452 10.1016/j.neuroimage.2010.03.049 20347043 PMC2872040

[B53] Negishi M. Martuzzi R. Novotny E. J. Spencer D. D. Constable R. T. (2011). Functional MRI connectivity as a predictor of the surgical outcome of epilepsy. Epilepsia 59, 1733–1740 10.1111/j.1528-1167.2011.03191.x 21801165 PMC3169719

[B54] Ogawa S. Lee T. M. Kay A. R. Tank D. W. (1990). Brain magnetic resonance imaging with contrast dependent on blood oxygenation. Proc. Natl. Acad. Sci. U.S.A. 87, 9868–9872 10.1073/pnas.87.24.9868 2124706 PMC55275

[B55] Ogawa S. Tank D. W. Menon R. Ellermann J. M. Kim S. G. Merkle H. (1992). Intrinsic signal changes accompanying sensory stimulation: functional brain mapping with magnetic resonance imaging. Proc. Natl. Acad. Sci. U.S.A. 89, 5951–5955 10.1073/pnas.89.18.8557 1631079 PMC402116

[B56] Ortega G. J. Menendez de la Prida L. Sola R. G. Pastor J. (2008a). Synchronization clusters of interictal activity in the lateral temporal cortex of epileptic patients: intraoperative electrocorticographic analysis. Epilepsia 49, 269–280 10.1111/j.1528-1167.2007.01266.x 17825075

[B57] Ortega G. J. Sola R. G. Pastor J. (2008b). Complex network analysis of human ECoG data. Neurosci. Lett. 447, 129–133 10.1016/j.neulet.2008.09.080 18848970

[B58] Palmigiano A. Pastor J. de Sola R. Ortega G. J. (2012). Stability of synchronization clusters and seizurability in temporal lobe epilepsy. PLoS ONE 7:e41799 10.1371/journal.pone.0041799 22844524 PMC3402406

[B59] Pereira F. R. Alessio A. Sercheli M. S. Pedro T. Bilevicius E. Rondina J. M. (2010). Asymmetrical hippocampal connectivity in mesial temporal lobe epilepsy: evidence from resting state fMRI. BMC Neurosci. 11:66 10.1186/1471-2202-11-S1-P66 20525202 PMC2890013

[B60] Pittau F. Grova C. Moeller F. Dubeau F. Gotman J. (2012). Patterns of altered functional connectivity in mesial temporal lobe epilepsy. Epilepsia 53, 1013–1023 10.1111/j.1528-1167.2012.03464.x 22578020 PMC3767602

[B61] Ponten S. C. Daffertshofer A. Hillebrand A. Stam C. J. (2010). The relationship between structural and functional connectivity: graph theoretical analysis of an EEG neural mass model. Neuroimage 52, 985–994 10.1016/j.neuroimage.2009.10.049 19853665

[B62] Ponten S. C. Douw L. Bartolomei F. Reikneveld J. C. Stam C. J. (2009). Indications for network regularization during absences seizures: weighted and unweighted graph theoretical analyses. Exp. Neurol. 217, 197–204 10.1016/j.expneurol.2009.02.001 19232346

[B63] Power J. D. Barnes K. A. Snyder A. Z. Schlaggar B. L. Petersen S. E. (2012). Spurious but systematic correlations in functional connectivity MRI networks arise from subject motion. Neuroimage 59, 2142–2154 10.1016/j.neuroimage.2011.10.018 22019881 PMC3254728

[B64] Quigley M. Cordes D. Wendt G. Turski P. Moritz C. Haughton V. (2001). Effect of focal and nonfocal cerebral lesions on functional connectivity studied with MR imaging. AJNR. Am. J. Neuroradiol. 22, 294–300 11156772 PMC7973944

[B65] Rogers B. P. Avery S. N. Heckers S. (2010). Internal representation of hierarchical sequences involves the default network. BMC Neurosci. 11:54 10.1186/1471-2202-11-54 20423509 PMC2868853

[B66] Rubinov M. Sporns O. (2010). Complex network measures of brain connectivity: uses and interpretations. Neuroimage 52, 1059–1069 10.1016/j.neuroimage.2009.10.003 19819337

[B67] Saini S. DeStefano N. Smith S. Guidi L. Amato M. P. Federico A. (2004). Altered cerebellar functional connectivity mediates potential adaptive plasticity in patients with multiple sclerosis. J. Neurol. Neurosurg. Psychiatry 75, 840–846 10.1136/jnnp.2003.016782 15145996 PMC1739042

[B68] Satterthwaite T. D. Elliott M. A. Gerraty R. T. Ruparel K. Loughead J. Calkins M. E. (2013). An improved framework for confound regression and filtering for control of motion artifact in the preprocessing of resting-state functional connectivity data. Neuroimage 64, 240–256 10.1016/j.neuroimage.2012.08.052 22926292 PMC3811142

[B69] Satterthwaite T. D. Wolf D. H. Loughead J. Ruparel K. Elliott M. A. Hakonarson H. (2012). Impact of in-scanner head motion on multiple measures of functional connectivity: relevance for studies of neurodevelopment in youth. Neuroimage 60, 623–632 10.1016/j.neuroimage.2011.12.063 22233733 PMC3746318

[B70] Schafer R. J. Lacadie C. Vohr B. Kesler S. R. Katz K. H. Schneider K. C. (2009). Alterations in functional connectivity for language in prematurely born adolescents. Brain 132(Pt 3), 661–670 10.1093/brain/awn353 19158105 PMC2664451

[B71] Scheinost D. Benjamin J. Lacadie C. M. Vohr B. Schneider K. C. Ment L. R. (2012). The intrinsic connectivity distribution: a novel contrast measure reflecting voxel level functional connectivity. Neuroimage 62, 1510–1519 10.1016/j.neuroimage.2012.05.073 22659477 PMC3538880

[B72] Schevon C. A. Cappell J. Emerson R. Isler J. Grieve P. Goodman R. (2007). Cortical abnormalities in epilepsy revealed by local EEG synchrony. Neuroimage 35, 140–148 10.1016/j.neuroimage.2006.11.009 17224281 PMC1994936

[B73] Shattuck D. W. Mirza M. Adisetiyo V. Hojatkashani C. Salamon G. Narr K. L. (2008). Construction of a 3D probabilistic atlas of human cortical structures. Neuroimage 39, 1064–1080 10.1016/j.neuroimage.2007.09.031 18037310 PMC2757616

[B74] Shehzad Z. Kelly A. M. Reiss P. T. Gee D. G. Gotimer K. Uddin L. Q. (2009). The resting brain: unconstrained yet reliable. Cereb. Cortex 19, 2209–2229 10.1093/cercor/bhn256 19221144 PMC3896030

[B75] Shen X. Papademetris X. Constable R. T. (2010). Graph-theory based parcellation of functional subunits in the brain from resting-state fMRI data. Neuroimage 50, 1027–1035 10.1016/j.neuroimage.2009.12.119 20060479 PMC3062848

[B76] Shen X. Tokoglu F. Papademetris X. Constable R. T. (2013). Groupwise whole-brain parcellation from resting-state fMRI data for network node identification. Neuroimage (in press). 10.1016/j.neuroimage.2013.05.081 PMC3759540 23747961

[B77] Stufflebeam S. M. Liu H. Sepulcre J. Tanaka N. Buckner R. L. Madsen J. R. (2011). Localization of focal epileptic discharges using functional connectivity magnetic resonance imaging. J. Neurosurg. 114, 1693–1697 10.3171/2011.1.JNS10482 21351832 PMC3248962

[B78] Towle V. L. Carder R. K. Khorasani L. Lindberg D. (1999). Electrocorticographic coherence patterns. J. Clin. Neurophysiol. 16, 528–547 10.1097/00004691-199911000-00005 10600021

[B79] Tzourio-Mazoyer N. Landeau B. Papathanassiou D. Crivello F. Etard O. Delcroix N. (2002). Automated anatomical labeling of activations in SPM using a macroscopic anatomical parcellation of the MNI MRI single-subject brain. Neuroimage 15, 273–289 10.1006/nimg.2001.0979 11771995

[B80] van Dellen E. Douw L. Baayen J. C. Heimans J. J. Ponten S. C. Vandertop W. P. (2009). Long-term effects of temporal lobe epilepsy on local neural networks: a graph theoretical analysis of corticography recordings. PLoS ONE 4:e8081 10.1371/journal.pone.0008081 19956634 PMC2778557

[B81] van den Heuvel M. Mandl R. Pol H. H. (2008). Normalized cut group clustering of resting-state fMRI data. PLoS ONE 3:e2001 10.1371/journal.pone.0002001 18431486 PMC2291558

[B82] Van Dijk K. R. Sabuncu M. R. Buckner R. L. (2012). The influence of head motion on intrinsic functional connectivity MRI. Neuroimage 59, 431–438 10.1016/j.neuroimage.2011.07.044 21810475 PMC3683830

[B83] Varotto G. Tassi L. Franceschetti S. Spreafico R. Panzica F. (2012). Epileptogenic networks of type II focal cortical dysplasia: a stereo-EEG study. Neuroimage 61, 591–598 10.1016/j.neuroimage.2012.03.090 22510255

[B84] Vincent J. L. Patel G. H. Fox M. D. Snyder A. Z. Baker J. T. Van Essen D. C. (2007). Intrinsic functional architecture in the anaesthetized monkey brain. Nature 447, 83–86 10.1038/nature05758 17476267

[B85] Waites A. B. Briellmann R. S. Saling M. M. Abbott D. F. Jackson G. D. (2006). Functional connectivity networks are disrupted in left temporal lobe epilepsy. Ann. Neurol. 59, 335–343 10.1002/ana.20733 16404743

[B86] Wang F. Kalmar J. H. Jackowski M. Chepenik L. G. Edmiston E. E. Tie K. (2009). Functional and structural connectivity between perigenual anterior cingulated and amydala in bipolar disorder. Biol. Psychiatry 66, 516–521 10.1016/j.biopsych.2009.03.023 19427632 PMC2830492

[B87] Wasserman S. Faust K. (1994). Social Network Analysis: Methods and Application. Cambridge: Cambridge University Press

[B88] Weissenbacher A. Kasess C. Gerstl F. Lanzenberger R. Moser E. Windischberger C. (2009). Correlations and anticorrelations in resting-state functional connectivity MRI: a quantitative comparison of preprocessing strategies. Neuroimage 47, 1408–1416 10.1016/j.neuroimage.2009.05.005 19442749

[B89] Widjaja E. Zamyadi M. Raybaud C. Snead O. C. Smith M. L. (2013). Impaired default mode network on resting-state fMRI in children with medically refractory epilepsy. Am. J. Neuroradiol. 34, 552–557 10.3174/ajnr.A3474 22954741 PMC7964920

[B90] Zhang X. Tokoglu F. Negishi M. Arora J. Winstanley S. Spencer D. D. (2011a). Social network theory applied to resting-state fMRI connectivity data in the identification of epilepsy networks with iterative feature selection. J. Neurosci. Methods 199, 129–139 10.1016/j.jneumeth.2011.04.020 21570425 PMC3129815

[B91] Zhang Z. Liao W. Chen H. Mantini D. Ding J.-R. Xu Q. (2011b). Altered functional-structural coupling of large-scale brain networks in idiopathic generalized epilepsy. Brain 134, 2912–2928 10.1093/brain/awr223 21975588

